# A Great Medical Discovery

**Published:** 1855-09

**Authors:** 


### A GREAT MEDICAL DISCOVERY.

### MERCURY TAKEN FROM THE SYSTEM BY ELECTRICITY.

The following is taken from a Western paper:

The following will be received with intense interest in every community where suffering of any kind is produced by metallic substances being introduced into the system in the way of mercury, gold, silver, or lead. If it is practically true, as scarcely any one can doubt under the circumstances, it is destined to rank among the greatest discoveries that science has yet brought to light.

The article which follows, published in the Scientific Bulletin of Paris, is entitled "The Application of Chemical Electricity to Therapeutics," and has been translated for this paper. Though not the literal, the substance of the article is intact. The Bulletin says:

Chemistry is about to drag from an anticipated death thousands of men, who, in the exercise of their cruel professions—gilding, looking-glass plating, white lead manufacturing, &c., and also those whose systems have been ruined by mercury in its various forms—for these science has raised her right arm and arrests their misery and destruction. This discovery extracts from their bodies, atom by atom, every particle of metallic substance from every part of the human system. Where do we get this great hope? In a memoir presented to the French Academy of Sciences by M. Dumas, which has for its authors two men, whose names will strike the ear of the public for the first time to-day. But if they prove what they promise to, they will soon take rank among the greatest benefactors of humanity. These authors are Andre Poly, of Havana, and Maurice Vergenes. The invention consists of an application of chemical electricity to accomplish the above purpose; and of all the marvellous things that electricity has achieved, this is the boldest and most triumphant.

The modus operandi is as follows:

A metallic bath is insulated from everything, and partially filled with acidulated water, to convey more readily the electrical currents. The patient lies upon a seat in the tub insulated entirely from the bath. When gold, silver, or mercury is in the system, nitric or hydrochloric acids are employed. When lead is suspected the acid used is sulphuric. This done, the negative pole of a battery is put in connection with the bath, while the positive pole is in the hands of the patient. Now the work of purification commneces. The electricity precipitates itself, hunts, digs, searches, and discovers every particle of metallic substance concealed in the most profound tissues, bones, joints, and nerves of the patient, resolves them into their primitive forms, and extracting them entire from the human organism, desposites them upon the sides of the bath, where they can be seen with the naked eye.

After the end of one of these operations, a chemist of Havana, M. Mossand, having analized 912 drachms of the liquid in the bath, he saw forming a metallic globule of the diameter of nine-tenths of a milimetre, and this was mercury. At another time the same chemist saw a very light white precipitated substance, which gave two globules of metallic lead perfectly visible to the naked eye, and M. Poly announced that he had taken from the tibia and thigh bone of a patient a quantity of mercury that had been there creating intense suffering for fifteen years.

Providence has had its usual hand in this discovery. One of the inventors, M. Maurice Vergenes, who was engaged at times in electric gilding, silvering, &c., where his hands came in continued contact with the nitrate and cyanuret of gold and silver, had them covered with ulcers, caused by particles of the metal being introduced into his blood, and no medical skill could eradicate them. One day he dipped his hands into the bath, taking hold of the positive pole of the battery, and at the end of fifteen minutes, to the surprise of the bystanders, a metallic plate of 163 milimetres in length by 109 in width placed in connection with the negative pole of the battery, was instantly covered with a thick coat of gold and silver extracted from his hands. The discovery was made. This event took place April 16, 1852. The inventors use 30 couples or batteries, or Bunson's and Grove's combined, it being found that a more energetic current will be evolved by this combination than by the use of either singly. Each couple is 40 milimetres in diameter by 217 in height. The number of these couples or batteries used at the commencement of an application, so as not to cause too much suffering for the patient, depends altogether upon the temperament of the patient and the nature of the disease. For example, a very nervous and delicate person would be submitted to the action of ten or twelve couples at first—the number increased at the rate of five couples every five minutes. A person of sanguine or lympathic temperament can endure more. The same ratio applies to the quality of acid in forming the bath; for instance it takes less for a nervous person than for a person with lymphatic or sanguine temperament. The metallic particles extracted from the body of the patient are deposited on the whole surface of the bathing tub, although the metal is formed in larger quantities opposite those parts of the body in which the metal lay concealed. As to the size of the metallic spots which are thus formed by the application of this discovery, they vary in size from that of the head of a pin to the size of a pea, and some are microscopic.

"I have seen," says M. Poly, "after the first bath of a person who had been complaining of tderrible pains in his arms, caused by mercury, the exact shape of the arm imprinted on the negative plate of the battery—the deposit being formed entirely of mercury drawn from the arm."

Here ends this most important article, which, if true, is destined to become as much a part of the medical practice as vaccination.—*Stethoscope.*

